# Associations of Gastrointestinal Tract Tumor Necrosis Factor Receptor-Associated Factor 6 Expression with Clinical Features and Prognosis of Eosinophilic Gastroenteritis

**DOI:** 10.5152/tjg.2023.22018

**Published:** 2023-06-01

**Authors:** Yaya Bai, Yuming Tang, Ying Zhu, Fei Yuan, Haimin Xu, Weiyan Yao

**Affiliations:** 1 Department of Gastroenterology, Ruijin Hospital, Shanghai Jiaotong University School of Medicine. Shanghai, China; 2 Department of Pathology, Ruijin Hospital, Shanghai Jiaotong University School of Medicine. Shanghai, China

**Keywords:** Eosinophilic gastroenteritis, tumor necrosis factor receptor-associated factor 6, pathology, prognosis

## Abstract

**Background:**

Few studies have been conducted to explore the expression of tumor necrosis factor receptor-associated factor 6 in eosinophilic gastroenteritis patients. Therefore, the expression profile of tumor necrosis factor receptor-associated factor 6 in the gastrointestinal tract of eosinophilic gastroenteritis patients and its associations with clinical features were explored in this study.

**Methods:**

Thirty-four eosinophilic gastroenteritis patients who presented in Ruijin Hospital from December 2012 to May 2019 and had accepted gastrointestinal endoscopic examinations were recruited. Medical records and endoscopic biopsies were collected, and the prognosis was followed up by telephone. Healthy persons were selected as the control group. Hematoxylin and eosin and immunohistochemical staining were performed in both eosinophilic gastroenteritis patients and healthy persons. The final results were analyzed by skilled pathologists, and mean optical density values of tumor necrosis factor receptor-associated factor 6 were calculated by Image J software. Final results were analyzed by Statistical Package for the Social Sciences software 22.0.

**Results:**

Thirty-four patients (mean age: 25.56 ± 21.14 years, 61.76% males) were recruited for this study. There was no significant difference in tumor necrosis factor receptor-associated factor 6 mean optical density values of gastric tissues in eosinophilic gastroenteritis patients and healthy people (0.22 ± 0.16 vs. 0.14 ± 0.05, *P *> .05). Eosinophilic gastroenteritis patients had a significantly lower level of intestinal tumor necrosis factor receptor-associated factor 6 mean optical density values than that of healthy people (0.16 ± 0.05 vs. 0.23 ± 0.06, *P *< .05). Intestinal tumor necrosis factor receptor-associated factor 6 mean optical density values negatively linearly correlated with serum interleukin-10 level (*r* = −0.618, *P*  = .043 < .05). There were no differences between eosinophilic gastroenteritis patients with or without relapse regarding the expression level of intestinal tumor necrosis factor receptor-associated factor 6 (*P*  = .227 > .05).

**Conclusion:**

Patients with eosinophilic gastroenteritis might have a deficiency of intestinal tumor necrosis factor receptor-associated factor 6 compared to healthy controls.

**Main Points**

- Eosinophilic gastroenteritis (EGE) is a chronic digestive disease that is related to food allergy, and with abdominal pain as the most commonly seen clinical manifestation, EGE is more prior to onset in young and middle-aged people.
- Eosinophil infiltration is the main pathological characteristic of EGE, and eosinophil counts are different in each part of the gastrointestinal tract.
- Tumor necrosis factor receptor-associated factor 6 (TRAF6) expression is positively expressed by brownish-yellow precipitates in the cytoplasm of epithelial cells and interstitial lymphocytes in gastrointestinal tissues of EGE patients.
- The lower expression level of intestinal TRAF6 of EGE patients is negatively correlated with peripheral interleukin 10, as well as the symptom duration of EGE patients.

## Introduction

Eosinophilic gastroenteritis (EGE) together with eosinophilic esophagitis (EoE), eosinophilic gastritis (EG), eosinophilic enteritis (EE), and eosinophilic colitis (EC) constitutes eosinophilic gastrointestinal diseases (EGIDs).^1^ While potential mechanisms of EoE have been discussed by many studies,^2,3^ the etiology and pathogenesis of EGE are still unclear. Besides, with unspecific clinical manifestations, laboratory or endoscopic findings, and an unidentified cutoff count of pathological criteria, EGE is still very difficult to be diagnosed clinically. Moreover, with a chronic and recurrent tendency, EGE still lacks unified management strategies. Glucocorticoid (GC) has been used in EGE patients as the first-line therapy, but a variety of adverse effects have shown up as great challenges when it comes to the maintenance treatment for patients with recurrence.^4^ For this reason, an urgent demand for new therapeutic targets to improve the diagnosis and treatment status of EGE makes research on pathogenesis and treatment targets of EGE become focused gradually.

It has been commonly believed that EGE might be related to food allergy in the past,^5^ but recent studies have shown that it is more likely related to immune-inflammatory responses involving T helper 2 cells (Th2).^6^ Tumor necrosis factor receptor-associated factor 6 (TRAF6) is a key adapter protein on the intracellular signaling pathway, contributing to maintaining the immune homeostasis of the gastrointestinal (GI) tract.^7^ Researchers have found that dendritic cell (DC)-specific deletion of TRAF6 could lead to the depletion of intestinal mucosal immune tolerance, manifested as spontaneous Th2 cell infiltration in the mucosal lamina propria, EE, and small intestinal fibrosis, which was probably correlated with the phenotype of human EGIDs,^8^ but until now, few studies have been conducted to explore its expression in human patients with EGE.

For the first time, this study explored the expression profiles of TRAF6 in the GI tract of EGE patients, and by collecting the clinical data and following up on the prognosis by telephone, clinical features that correlated with TRAF6 have also been analyzed for the purpose of exploring potential diagnostic markers of EGE.

## Materials and Methods

All the physicians involved in the study followed the ethics guidelines for human medical research according to Helsinki declaration of 1975, and this study was approved by the ethics committee of Ruijin Hospital affiliated to Shanghai Jiaotong University (no: 2019-162). Patients in the experimental and the control group both signed informed consent.

### Patients Enrollment

Thirty-four cases diagnosed with EGE in Ruijin Hospital affiliated to Shanghai Jiaotong University from October 2012 to May 2019 were analyzed retrospectively. All the patients were included based on Talley’s diagnostic criteria of EGE^4^ which included the following: (1) the presence of GI symptoms, (2) biopsies showing eosinophilic infiltration of 1 or more areas of the GI tract from esophagus to colon or characteristic radiological findings with peripheral eosinophilia, and (3) no evidence of parasitic or extraintestinal disease, such as connective tissue disease, eosinophilia, Crohn’s disease, primary lymphoma, amyloidosis, and Menetrier’s disease. Patients were excluded based on the following criteria: severe lack of medical records; the diagnosis of EGE being unverified; and having suffered from parasitic infection, connective tissue disease, malignant tumor, inflammatory bowel disease (IBD), and other diseases. All the endoscopy reports and pathology reports of those 34 cases were collected, as well as the medical records, lab test results, radiological images and even test results of parasitic detection, bone marrow punctures, and abdominal punctures were scrutinized and summarized for the differential diagnosis of EGE. Recurrent profiles and GC treatment status were followed up by telephone.

**Figure 1.**
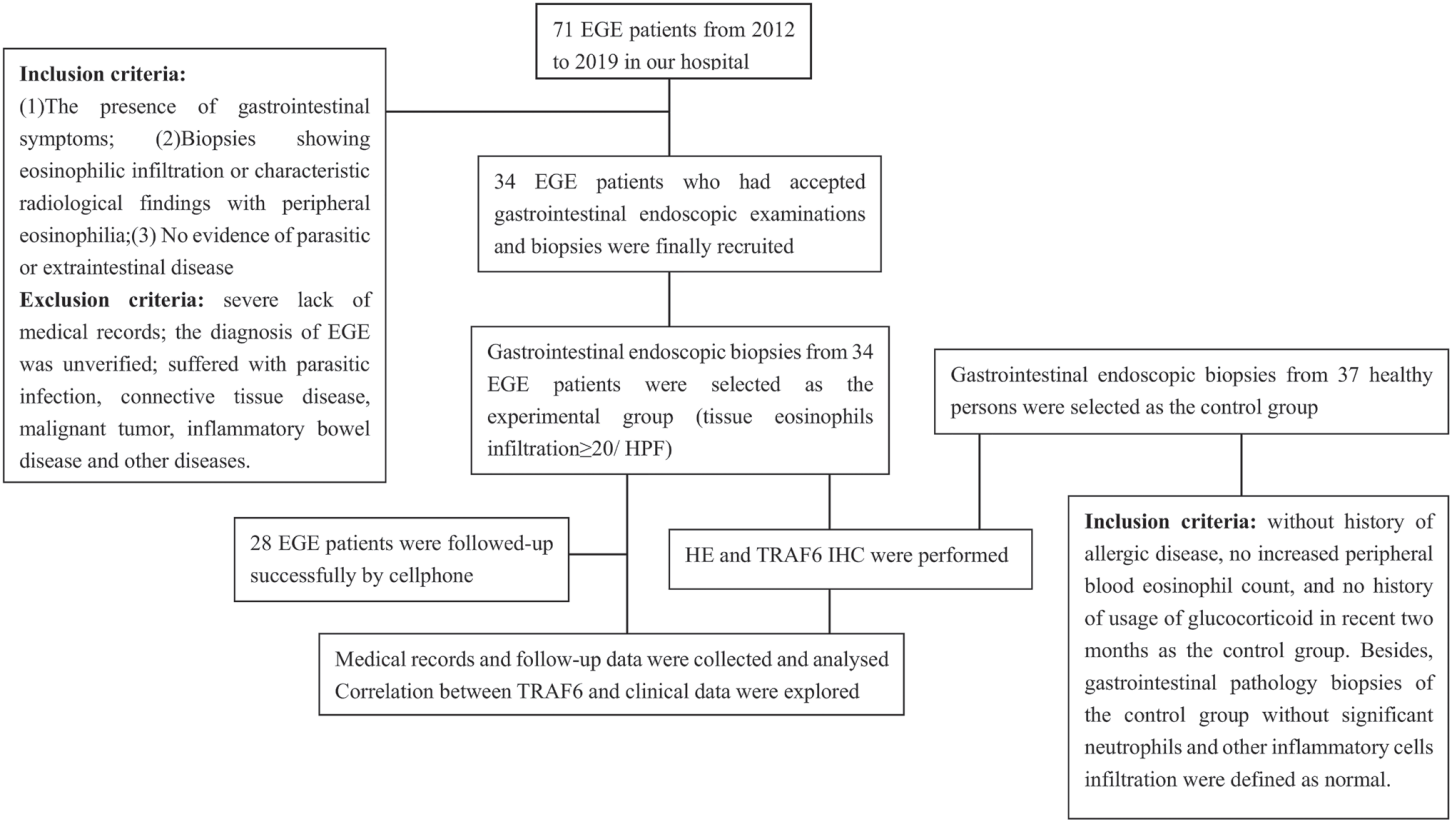
Flowchart of research process.

Healthy people who underwent health checkups, including endoscopic procedures with tissue biopsies in Ruijin Hospital, were selected as the control group in this study. According to the routine of our hospital, some people underwent endoscopy examinations and tissue biopsies to confirm the normal state. Therefore, this study selected patients without a history of allergic disease, with no increased peripheral blood eosinophil count, and with no history of usage of GC in recent 2 months as the control group. Besides, GI pathology biopsies of the control group without significant neutrophils and other inflammatory cells infiltration were defined as normal (Figure 1).

### Tissue Samples

Gastrointestinal tissue eosinophil infiltration ≥20/high power field (HPF) was defined as pathologically positive in EGE patients.^9^ Based on this criteria, 34 EGE patients were included in this study as the experimental group; of which, 15 patients had accepted upper GI endoscopic examination (n = 15), while 22 patients had accepted lower GI endoscopic examination (n = 22). Biopsies from healthy people were selected as the control group according to the matching principle of 1:1 (gastric tissues, n = 15; intestinal tissues, n = 22). Hematoxylin and eosin staining and IHC staining were performed in both experimental and control groups to compare the pathological characteristics and the expression levels of TRAF6. Then, the results were analyzed by skilled pathologists, and mean optical density (MOD) values of TRAF6 were calculated by ImageJ software (ImageJ 1.52a, Wayne Rasband, National Institutes of Health, USA).

### Hematoxylin and Eosin and Immunohistochemical Staining

Biopsy tissues were immersed in neutral formalin at 4°C for 24 hours and embedded with paraffin. Serial 4-6 µm sections were obtained from each block, with the first slide stained with H&E to confirm the pathological diagnosis, and subsequent slides were stained for further IHC staining. Tissue slides were routinely deparaffinated and rehydrated. Heat-mediated antigen retrieval with citrate buffer of pH 7.2 was performed before commencing with the IHC staining protocol. After antigen retrieval (Shanghai Lai Zi Biotechnology, China) and blocking with rabbit immune serum (Shanghai Lai Zi Biotechnology, China), the sections were incubated with the monoclonal antibody against TRAF6 (1:75 dilutions; ab33915; Abcam, Cambridge, Mass, USA) in a 37°C incubator for 2 hours, washed with phosphate buffered saline (PBS), incubated with secondary antibody for 1 hour, washed again with PBS, and incubated with horseradish peroxidase-labeled streptavidin (Shanghai Lai Zi Biotechnology, China) for another 15 minutes. Afterward, the sections were washed with PBS, colored with diaminobenzidine (DAB) (Shanghai Lai Zi Biotechnology, China), and observed under a microscope. PBS was used to replace the primary antibody as the negative control. With regard to standards for the determination of IHC staining, TRAF6 was determined to be positively expressed in the case of brownish-yellow precipitates in the cytoplasm. The procedure was completed by 2 experienced pathologists.

### Semiquantitative Analysis of Tumor Necrosis Factor Receptor-Associated Factor 6

The sections were photographed Carl Zeiss Image (CZI) under exactly the same conditions, and images were processed uniformly with Zen Blue Lite (ZEN 2.3(blue edition), Carl Zeiss Microscopy Gmbh,2011). The semiquantitative analysis, calculation of MOD value of TRAF6, was carried out by ImageJ software (ImageJ 1.52a, Wayne Rasband), and the average value was calculated 3 times for each sample.

### Statistical Analysis

All analyses were performed using Statistical Package for the Social Sciences software version 22.0 for Windows (IBM Corp.; Armonk, NY, USA). A 2-sided *P* < .05 was considered statistically significant. Quantitative and qualitative parameters were presented as mean ± standard deviation and numbers with the percentage in parentheses, compared among groups by Student’s *t*-test, nonparametric rank sum test, 1-way analysis of variance, or chi-squared test, respectively. Pearson’s correlation analysis was applied to investigate the correlation between TRAF6 and clinical data.

## Results

### Baseline Characteristics of Study Population

Thirty-four patients (mean age: 25.56 ± 24.14 years, 61.76% males) were recruited for this study. Among them, 13.33% of the patients had a smoking history, and 9.68% of the patients had a drinking history. Of 34 patients, 8.82% had family allergic history and 44.12% had allergic history; of which, food allergy took a proportion of 60.0%, and eggs were the most common in food allergies (55.56%). Most EGE patients had no obvious predisposition factors (50.0%), while food-related factors were the second leading cause (44.12%) in our study. Eosinophilic gastroenteritis patients had no specific clinical symptoms, while abdominal pain was the most common one (70.59%). About 58.82% of the patients had a symptom duration of over half a year which probably was accordant with the chronic tendency of EGE. There were 3 types of EGE: mucosal type (91.18%), muscular type (2.94%), and serosal type (5.88%). Eosinophilic gastroenteritis patients in our study had an elevated level of peripheral blood eosinophils (3.46 ± 3.87 × 10^9^/L) and eosinophils direct count (2.56 ± 2.63 × 10^9^/L), as well as serum immunoglobulin E (289.74 ± 465.85 IU/mL). Some patients had an elevation of erythrocyte sedimentation rate and C-reactive protein. The mean value of interleukin (IL)-2, IL-6, IL-10, and tumor necrosis factor were 684.42 ± 352.56 pg/mL, 6.39 ± 4.30 pg/mL, 8.03 ± 6.50 pg/mL, and 19.04 ± 21.34 pg/mL, respectively. Of 34 EGE patients, 67.86% accepted GC treatment and 57.14% of patients had a relapse; of which, multiple recurrence type was the most common type (68.75%) (Table 1).

### Endoscopic Examinations of Eosinophilic Gastroenteritis Patients

There were 85.29% patients who accepted gastroscopic examination and 86.21% who accepted colonoscopic examination. As previous studies reported that EGE had no specific endoscopic manifestations, in our study, EGE patients had nonspecific manifestations including erythema, congestion and edema, erosion, ulcer, and polyps (Table 1). As for endoscopic biopsies, the number of biopsy sites for gastric and intestinal endoscopy was 2.19 ± 1.00 and 2.68 ± 1.79 respectively, while the number of gastric or intestinal biopsy tissue blocks was 3.19 ± 2.04 and 3.93 ± 2.75, respectively. Pathological eosinophil counts of gastric and intestinal biopsies were 21.93 ± 14.13/HPF and 31.32 ± 16.98/HPF. In addition, we displayed the pathological eosinophil counts comprehensively in different parts of the GI tract of EGE patients (Table 2).

### Baseline Clinical Characteristics of Eosinophilic Gastroenteritis Patients and the Control Group

The mean age of EGE patients who accepted gastric biopsy was 26.40 ± 18.86 years, and the ratio of male to female was 9:6. In the control group, the average age was 46.90 ± 21.14 years, and the ratio of male to female was 6:9; there was no significant difference as for the mean age and sex composition ratio of the experimental group and the control group (*P* > .05). The mean age of EGE patients who accepted intestinal biopsy was 20.59 ± 23.88 years, and the ratio of male to female was 17:5. In the control group, the average age was 22.41 ± 21.27 years, the male–female ratio was 16:6; there was no significant difference in age and sex ratio between the experimental and control group (*P* > .05) (Table 3).

### Pathologic Features and Tumor Necrosis Factor Receptor-Associated Factor 6 Expression Profiles of Eosinophilic Gastroenteritis Patients and the Control Group

**Figure 2.**
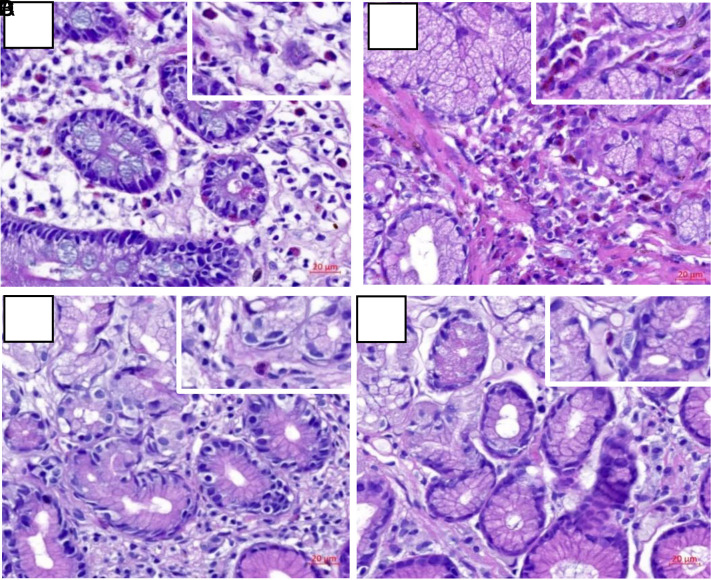
H&E staining of gastric biopsy tissues in the experimental and control group 10× (magnification times regionally 20×) (A-B) Experimental group of EGE. (A) Eosinophil infiltration in the interstitium of gastric tissues. (B) More eosinophil infiltration accompanied with the increase of lymphocytes in the interstitium of gastric tissues. (C-D) Control group of normal persons. (C) Few eosinophil infiltration in the interstitium of gastric tissues. (D) Fewer lymphocytes in the interstitial tissues compared to the experimental group. H&E, hematoxylin and eosin; EGE, eosinophilic gastroenteritis.

**Figure 3.**
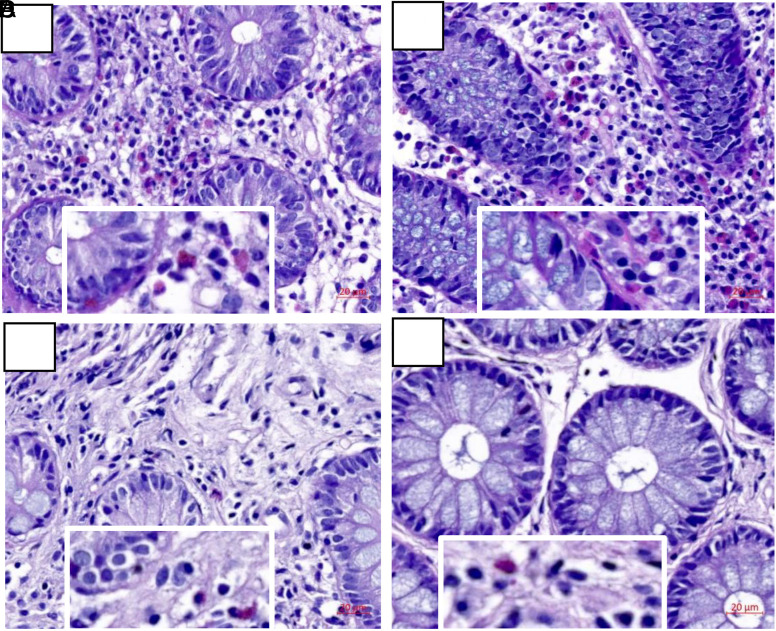
H&E staining of intestinal biopsy tissues in the experimental and control group 10× (magnification times regionally 20×) (A-B) Experimental group of EGE. (A) Eosinophil infiltration obviously. (B) Eosinophil infiltration accompanied with the increase of lymphocytes in the interstitium of intestinal tissues. (C-D) Control group of normal persons. (C) Only few eosinophil infiltration in the interstitium of intestinal tissues. (D) Fewer lymphocytes in the interstitial tissues compared to the experimental group. H&E, hematoxylin and eosin; EGE, eosinophilic gastroenteritis.

According to HE staining, eosinophil infiltration accompanied by increased lymphocytes could be found in the interstitium of gastric tissue samples of EGE patients more obviously than that of the control group, which was similar to the pathological features of eosinophil infiltration in the intestinal tissues, interstitial eosinophilia accompanied by increased lymphocytes (Figure 2). However, eosinophil and lymphocyte infiltrations were more obvious in the interstitium of intestinal tissues (Figure 3).

**Figure 4.**
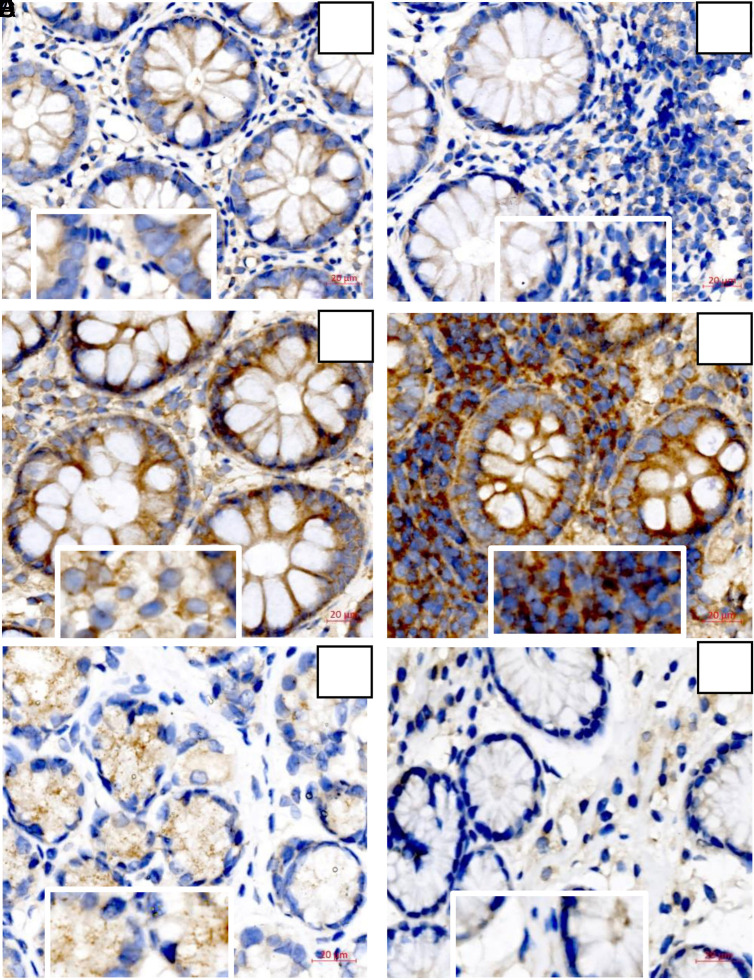
Immunohistochemical staining of TRAF6 of experimental and control group 10× (magnification times regionally 20×) (A-B) The staining of TRAF6 in the intestinal tissue of experimental group. (A) Cytoplasmic positive expression in intestinal mucosal epithelium and interstitial lymphocytes. (B) More lymphocytes infiltration accompanied with weaker positive expression of TRAF6 in the interstitium of intestinal tissues. (C-D) The staining of TRAF6 in the intestinal tissue of control group. (C) Strong positive expression of TRAF6 in cytoplasm of intestinal epithelium and lymphocytes. (D) Strong diffuse positive expression in intestinal epithelium accompanied with the strong positive expression in cytoplasm of interstitial lymphocytes. (E) The focal positive expression of TRAF6 in gastric mucosal epithelium and the weak positive expression of interstitial lymphocytes in the experimental group. (F) The epithelium and interstitium were weakly positive in the control group. TRAF6, tumor necrosis factor receptor-associated factor 6.

In terms of IHC staining, TRAF6 expression was positive in the cytoplasm of epithelial cells and interstitial lymphocytes in GI tissues of EGE patients, characterized by brownish-yellow precipitates. In gastric tissue samples of EGE patients, few eosinophils and lymphocytes could be found in the interstitium and TRAF6 expression was focal or weak positive in the epithelium cells, while in the control group, TRAF6 expression was even more weak positive in the epithelium of gastric tissue. In intestinal tissue samples, however, strong diffuse positive expression of TRAF6 in the cytoplasm of intestinal epithelium accompanied by the strong positive expression in the cytoplasm of interstitial lymphocytes was shown in the control group more obviously than that in the experimental group (Figure 4).

Gastric MOD values of TRAF6 were 0.22 ± 0.16 and 0.14 ± 0.05 in the experimental and control groups, respectively, and there was no statistical difference between the 2 groups (*P > *.05). Intestinal MOD values of TRAF6 were 0.16 ± 0.05 and 0.23 ± 0.06 in the experimental and the control groups, respectively, and the difference was statistically significant *(P *< 0.05) (Table 3).

### Correlations of Tumor Necrosis Factor Receptor-Associated Factor 6 and Clinical Data of Eosinophilic Gastroenteritis Patients

There was no significant difference in intestinal TRAF6 MOD values of EGE patients of different age, sex, different allergy history, food allergy history, positive condition of allergic test, and elevated level of peripheral blood eosinophils (*P > *.05, data were not shown). Eosinophilic gastroenteritis patients with different duration of symptoms before diagnosis had different levels of expression of TRAF6 in the intestinal tissues (*P*  = .011 < .05) (Table 4); among which, patients with a disease course over half a year had a significantly lower level of TRAF6 expression than those with a disease course less than 2 weeks (*P* = .028 < .05)(Table 5).

According to the follow-up study, there was no difference of TRAF6 expression in the intestinal tissues of patients with or without recurrence (*P* = .227 > .05).

In this study, EGE patients with elevated serum IL-10 had lower expression levels of intestinal TRAF6 than patients with normal serum IL-10 levels (*P* = .027 < .05). The MOD values of TRAF6 were negatively linear with serum IL-10 levels (*r* = −0.618, *P* = .043 < .05)(Table 6).

## Discussion

Eosinophilic gastroenteritis is a rare chronic disease of the digestive system with an unclear etiology, unspecific clinical manifestations, and an unidentified cutoff count of pathological diagnostic criteria, so it is very difficult to diagnose it currently. Recently, studies focusing on new diagnostic markers have become more and more crucial.^10^ Therefore, we conducted this study to explore the possibilities of TRAF6 to be a new preliminary diagnostic marker for EGE, hoping to contribute to the individualized management of EGE patients clinically.

Tumor necrosis factor receptor (TNFR)-associated factor 6 is an adapter protein that mediates a wide array of protein–protein interactions via its TRAF domain and a RING finger domain that possesses non-conventional E3 ubiquitin ligase activity.^7,11^ Tumor necrosis factor receptor-associated factor 6-mediated signals have proven critical for the development and homeostasis of the immune system. The human TRAF6 gene is located in 11p13, a coding protein with 511 amino acid residues.^12^ Physiologically, TRAF6 expresses moderately in the GI tissues, which is helpful for the maintenance of immune tolerance in the digestive system.^13^ Also, the TRAF6-mediated nuclear factor kappa-B signaling pathway is crucial for the maturation and development of intestinal M cells.^14^ Research has shown that the high expression level of TRAF6 in gastric cancer, esophageal cancer, and colorectal cancer is significantly related to the prognosis of these diseases.^15-17^ In this study, IHC staining was performed in the GI tissues in both EGE patients and healthy controls and then semiquantitative analysis was conducted by ImageJ software to confirm the MOD values of TRAF6. Finally, we found that patients with EGE had a significantly lower intestinal expression level of TRAF6 compared with healthy persons, and lower expression of TRAF6 in the intestinal tissues of EGE is negatively correlated with peripheral blood IL-10 levels and the duration of symptoms.

The etiology of EGE is still unclear, but it is currently thought to be an allergic and immune-inflammatory disease.^18^ Several studies have revealed that TRAF6 is related to various allergic and immune-related diseases, such as asthma, systemic lupus erythematosus (SLE), rheumatoid arthritis (RA), IBD,^19-21^ and so on. Nowadays, it has been reported by researchers that the deficiency of TRAF6 in DCs can induce eosinophil infiltration in the intestinal tissues of mice, and this genotype is very likely to have a connection with the phenotype of EGID in the human body.^8^ However, there was no research to explore the expression of TRAF6 in patients with EGE until now. So we conducted this study to explore the expression of TRAF6 in EGE patients through IHC staining and analyzed its correlations with the clinical features and prognosis of EGE for the first time.

Interleukin-10 was originally purified from activated CD4+ T helper (Th) 2 cells named cytokine synthesis inhibitory factor).^22^ Compromised signaling of the IL-10 pathway is associated with inflammatory diseases such as IBD and is often accompanied by immunopathology during infections.^23^ Interleukin-10-producing B cells have been suggested to be aberrant in SLE or RA.^24,25^ Human genetic studies further supported IL-10 in controlling intestinal inflammation.

Eosinophilic gastroenteritis is dominated by Th2 immune response, and previously several studies demonstrated that various Th2 cytokines such as IL-5, IL-15, IL-13, IL-33, and thymic stromal lymphopoietin were related to EGE,^26-28^ but few research have ever illustrated the expression profile of IL-10 in EGE. In our study, we found that lower expression of TRAF6 in the intestinal tissues of EGE is negatively correlated with peripheral blood IL-10 levels, which probably indicates that the deficiency of TRAF6 in the intestinal tissues of patients with EGE tends to afflict the mucosal barrier of GI tract and then causes a Th2-dominated immune response which is closely related to the higher expression of peripheral IL-10. This is the first time for IL-10 to be found to correlate with the immune-inflammatory response of EGE in the background of lower expression of TRAF6, which pave the way for further studies dedicated to finding new diagnostic markers of EGE.

According to the follow-up study, we found that lower expression of TRAF6 in the intestinal tissues of EGE is negatively correlated with the duration of symptoms. In Pineton’s study,^29^ EGE was classified as mucosal, subserosal, or muscular type in 44%, 39%, and 12% of cases, respectively. After a median follow-up period of 13 years, 3 different courses of disease progression were identified: 18 patients had an initial flare of the disease without relapse, 16 had multiple flares that were separated by periods of full remission, and 9 had chronic courses. In our study, there was no significant difference in MOD values of EGE patients with or without recurrence. The duration of the symptom was classified into 2 weeks, 2 weeks to half a year, and over half a year, and then it was found that patients with a longer duration of symptom had a lower expression level of TRAF6 in the intestinal tissues. Previously, no research had ever found that TRAF6 expression in the intestinal of EGE patients correlated with its course. We predicate that the lower expression of TRAF6 in the intestines of EGE patients might contribute to the chronic course of EGE.

However, there are some limitations to our study. Firstly, as a cross-sectional study with small sample size, selection bias might occur and the results need to be further confirmed in prospective studies. Secondly, although we pre-experimentally screened several molecules and finally selected TRAF6, co-expression of the relevant molecules should be further investigated. In addition, although there was no significant difference as for the mean age of EGE patients and healthy controls who had accepted gastric biopsy (26.40 ± 18.86 vs. 46.90 ± 21.14, *P* > .05), the prevalence of either chronic atrophic gastritis or chronic non-atrophic gastritis increased with age,^30^ while different backgrounds of tissue inflammation may have different effects on the experimental results. Therefore, even though the age difference was not statistically significant, its influence on the results should be taken into consideration in our study because of the small sample size. Finally, we only did IHC at the protein level, while further molecular signaling pathways should be deeply explored to elaborate the mechanisms of EGE.

In conclusion, patients with EGE might have a deficiency of intestinal TRAF6 compared to healthy controls. Lower expression of intestinal TRAF6 might suggest an elevated level of serum IL-10 and a longer duration of symptoms in EGE patients.

## Figures and Tables

**Table 1.** Baseline Characteristics

Variables	Mean ± SD, %	Variables	Mean ± SD, %
Age (years)	25.56 ± 24.14	Symptom duration	
Sex (males, %)	61.76% (21/34)	≤2 weeks	17.65% (6/34)
Height (cm)	142.27 ± 29.53	2 weeks to half a year	23.53% (8/34)
BMI (kg/m^2^)	19.13 ± 5.10	Greater than or equal to half a year	58.82% (20/34)
Smoking history (%)	13.33% (4/30)	EGE types (%)	
Drinking history (%)	9.68% (3/31)	Mucosal type	91.18% (31/34)
Allergic history (%)	44.12% (15/34)	Muscular type	2.94% (1/34)
Asthma (%)	20.0% (3/15)	Serosal type	5.88% (2/34)
Anaphylactic rhinitis (%)	20.0% (3/15)	Peripheral eosinophils (×10^9^/L)	3.46 ± 3.87
Allergic dermatitis (%)	6.67% (1/15)	EOS direct count (×10^9^/L)	2.56 ± 2.63
Drug allergy (%)	13.33% (2/15)	ESR (mm/s)	6.00 (2.75, 13.25)
Urticaria (%)	13.33% (2/15)	CRP (mg/L)	1.00 (0.21, 11.71)
Nasal polyp (%)	6.67% (1/15)	IgE (IU/mL)	289.74 ± 465.85
Food allergy(%)	60.0% (9/15)	IL-2 (pg/mL)	684.42 ± 352.56
Milchigs (%)	44.44% (4/9)	IL-6 (pg/mL)	6.39 ± 4.30
Eggs (%)	55.56% (5/9)	IL-10 (pg/mL)	8.03 ± 6.50
Seafoods (%)	44.44% (4/9)	TNF (pg/mL)	19.04 ± 21.34
Grains (%)	33.33% (3/9)	Serum IgE stratification (IU/mL)	
Beans (%)	.-	≤100	25.96% (7/27)
Vegetables and fruits (%)	33.33% (3/9)	100-200	33.33% (9/27)
Family allergic history (%)	8.82% (3/34)	≥200	40.74% (11/27)
Hospitalization duration (days)	10.72 ± 6.87	Gastroscope examination (yes or no, %)	85.29% (29/34)
Predisposition factors (%)		Colonoscope examination (yes or no, %)	86.21% (25/29)
Food related	44.12% (15/34)	Erythema (gastric vs. intestinal, %)	11.76%, 9.09%
Season related	2.94% (1/34)	Congestion and edema (gastric vs. intestinal, %)	55.88%, 63.64%
Infection	2.94% (1/34)	Erosion (gastric vs. intestinal, %)	35.29%, 40.91%
No obvious inducement	50.00% (17/34)	Ulcer (gastric vs. intestinal,%)	20.59%, 31.82%
Symptoms (%)		Polyp (gastric vs. intestinal, %)	NA, 13.64%
Abdominal pain	70.59% (24/34)	Successfully followed up (%)	82.35% (28/34)
Abdominal distension	14.71% (5/34)	Glucocorticoid treatment (yes, %)	67.86% (19/28)
Nausea	11.76% (4/34)	Recurrent (yes, %)	57.14% (16/28)
Vomitting	26.47% (9/34)	Recurrent once (%)	18.75% (3/16)
Pyoperitoneum	2.94% (1/34)	Multiple recurrence (%)	68.75% (11/16)
Diarrhea	47.06% (16/34)	Continuous (%)	12.5% (2/16)
Fever	8.82% (3/34)	Gastric TRAF6 MOD value	0.21 ± 0.15
Hematochezia	17.65% (6/34)	Intestinal TRAF6 MOD value	0.16 ± 0.05

EGE, eosinophilic gastroenteritis; EOS, eosinophils; ESR, erythrocyte sedimentation rate; CRP, C-reactive protein; IgE, immunoglobulin E; IL-2, Interleukin 2; IL-6, Interleukin 2; IL-10, Interleukin 10; TNF, tumor necrosis factor; TRAF6, tumor necrosis factor receptor-associated factor 6; MOD, mean optical density; SD, standard deviation.

**Table 2.** Endoscopic Biopsy Status and Pathological Eosinophils

Endoscopic Examination	Number of Biopsy Sites	Number of Biopsy Tissue Blocks	Pathological EOS Count/HPF
	Mean ± SD	Mean ± SD	Mean ± SD
Gastroscopic biopsy	2.19 ± 1.00	3.19 ± 2.04	21.93 ± 14.13
Esophagus		1.17 ± 0.41	20.00 ± 0.00
Gastric antrum		1.54 ± 0.71	20.29 ± 17.91
Body of stomach		1.67 ± 1.21	N/A
Fundus of stomach		N/A	N/A
Angle of gastric		N/A	N/A
Duodenal bulb		1.14 ± 0.38	23.89 ± 11.67
Descending part of duodenum		1.44 ± 0.88	N/A
Colonoscopic biopsy	2.68 ± 1.79	3.93 ± 2.75	31.32 ± 16.98
Terminal ileum		1.67 ± 1.16	34.17 ± 18.55
Ileocecus		1.46 ± 1.13	31.00 ± 17.46
Ascending colon		1.38 ± 0.52	35.00 ± 15.00
Transverse colon		1.15 ± 0.38	23.75 ± 12.75
Descending colon		1.45 ± 0.52	22.86 ± 16.04
Sigmoid colon		1.31 ± 0.48	31.00 ± 23.02
Rectum		1.13 ± 0.35	14.00 ± 9.62

N/A, none; EOS, eosinophils; SD, standard deviation; HPF, high power field.

**Table 3.** Comparison of Baseline Characteristics and TRAF6 Expressions of EGE Patients and the Control Group

Items	Gastric		*P*	Intestinal		*P*
	Experimental	Control		Experimental	Control	
Mean age (years)	26.40 ± 18.86	46.90 ± 21.14	.724	20.59 ± 23.88	22.41 ± 21.27	.597
F : M ratio	9 : 06	6 : 09	.466	17 : 05	16 : 06	.728
Mean MOD	0.22 ± 0.16	0.14 ± 0.05	.089	0.16 ± 0.05	0.23 ± 0.06	<.001

TRAF6, tumor necrosis factor receptor-associated factor 6; MOD, mean optical density; EGE, eosinophilic gastroenteritis.

**Table 4.** TRAF6 Expression in Intestinal Tissues of EGE Patients

Variables	MOD of TRAF6 (mean ± SD)		*P*
Sex	0.16 ± 0.06 (male) vs. 0.15 ± 0.04 (female)		.573
Age	0.16 ± 0.07 (adults) vs. 0.16 ± 0.04 (children)		.946
Allergy history	0.15 ± 0.04 (yes) vs. 0.17 ± 0.05 (no)		.334
Food allergy	0.14 ± 0.03 (yes) vs. 0.17 ± 0.05 (no)		.334
Allergy tests	0.15 ± 0.05 (positive) vs. 0.15 ± 0.04 (negative)		.936
Eosinophils in peripheral blood	Normal	0.19 ± 0.04	.115
	Increased	0.15 ± 0.05	
	Decreased	0.16 ± 0.05	
Symptom duration	<2 weeks	0.24 ± 0.07	.011
	2 weeks to 6 months	0.16 ± 0.09	
	≥6 months	0.15 ± 0.04	
Recurrence	0.15 ± 0.04 (yes) vs. 0.18 ± 0.06 (no)		.227

SD, standard deviation; EGE, eosinophilic gastroenteritis; MOD, mean optical density; TRAF6, tumor necrosis factor receptor-associated factor 6.

**Table 5.** Comparison of TRAF6 MOD Value in Different Durations of Clinical Symptoms

Symptom Duration		Mean	SE	*P*	95% CI	
≤2 weeks	2 weeks to half year	0.076	0.044	.098	−0.015	0.168
	Greater than or equal to half a year	0.086^*^	0.036	.028	0.011	0.161
2 weeks to half year	≤2 weeks	−0.076	0.044	.098	-0.168	0.015
	Greater than or equal to half a year	0.010	0.030	.752	-0.053	0.073
Greater than or equal to half a year	≤2 weeks	−0.086	0.036	.028	−0.161	−0.011
	2 weeks to half year	−0.010	0.030	.752	−0.073	0.053

*Significant at .05 level.

MOD, mean optical density SE, standard error; TRAF6, tumor necrosis factor receptor-associated factor 6.

**Table 6.** Pearson’s Correlation of TRAF6 and Clinical Data

Variables		Age (years)	BMI (Kg/m^2^)	Hospitalization Duration (days)	Peripheral Eoxinophils (×10^9^/L)	EOS Direct Count (×10^9^/L)	ESR (mm/h)	CRP (mg/L)	IgE (IU/mL)	IL-2 (pg/mL)	IL-6 (pg/mL)	IL-10 (pg/mL)	TNF (pg/mL)	Gastric EOS Count/HPF	Intestinal EOS Count/HPF
Gastric TRAF6 MOD value	Pearson’s correlation	−0.168	0.173	0.006	−0.091	−0.091	−0.278	0.436	−0.541	−1.000^**^	−1.000^**^	^***^	−1.000^**^	0.421	0.893^*^
	*P*-value	.548	.538	.983	.747	.846	.315	.104	.133	-	-	-	-	.259	.041
Intestinal TRAF6 MOD value	Pearson’s correlation	0.184	0.111	−0.176	−0.138	0.348	−0.418	−0.315	0.123	−0.055	−0.487	−0.618^*^	−0.086	0.543	−0.142
	*P*-value	.425	.631	.446	.551	.652	.059	.164	.628	.874	.077	.043	.760	.208	.614

^*^Significant at the .05 level (2-tailed);

^**^Significant at the .01 level (2-tailed);

^***^Computing cannot be performed because at least 1 variable is a constant.

BMI, body mass index; ESR, erythrocyte sedimentation rate; CRP, C-reactive protein; IgE, immunoglobulin E; IL-2, interleukin 2; IL-6, interleukin 2; IL-10, interleukin 10; TNF, tumor necrosis factor; EOS, eosinophils; TRAF6, tumor necrosis factor receptor-associated factor 6; MOD, mean optical density; HPF, high power field.
